# Microcontact Printing of Proteins for Cell Biology

**DOI:** 10.3791/1065

**Published:** 2008-12-05

**Authors:** Keyue Shen, Jie Qi, Lance C. Kam

**Affiliations:** Department of Biomedical Engineering, Columbia University

## Abstract

The ability to pattern proteins and other biomolecules onto substrates is important for capturing the spatial complexity of the extracellular environment.  Development of microcontact printing by the Whitesides group (http://gmwgroup.harvard.edu/) in the mid-1990s revolutionalized this field by making microelectronics/microfabrication techniques accessible to laboratories focused on the life sciences.  Initial implementations of this method used polydimethylsiloxane (PDMS) stamps to create patterns of functionalized chemicals on material surfaces^1^.  Since then, a range of innovative approaches have been developed to pattern other molecules, including proteins^2^.  This video demonstrates the basic process of creating PDMS stamps and uses them to pattern proteins, as these steps are difficult to accurately express in words.  We focus on patterning the extracellular matrix protein fibronectin onto glass coverslips as a specific example of patterning. An important component of the microcontact printing process is a topological master, from which the stamps are cast; the raised and lowered regions of the master are mirrored into the stamp and define the final pattern.  Typically, a master consists of a silicon wafer coated with photoresist and then patterned by photolithography, as is done here.  Creation of masters containing a specific pattern requires specialized equipment, and is best approached in consultation with a fabrication center or facility.  However, almost any substrate with topology can be used as a master, such as plastic diffraction gratings (see Reagents for one example), and such serendipitous masters provide readily available, simple patterns.  This protocol begins at the point of having a master in hand.

**Figure:**
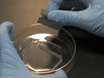


## Protocol

### 1. Preparation of solutions and materials

These steps should be carried out several days in advance.

1. Glass coverslips. Coverslips were cleaned by immersion for 10 minutes into a solution of Linbro 7X detergent : water, mixed at a 1 : 3 ratio and heated, with stirring, until clear.  Coverslips were rinsed extensively with deionized water, and then baked at 450°C for 6 hours.  Loading coverslips into ceramic staining racks (see Reagents) simplified this process.
2. Protein solution for stamping. Reconstitute fibronectin following the manufacturers instructions to a stock solution of 1 mg/ml concentration.

### 2. Cast stamps from the topological master

These steps can be carried out several days in advance. Store stamps pattern-up in a covered dish, such as a tissue culture dish.

1. Remove loose dust from master using a stream of compressed, filtered air or inert gas.
2. Place the master, patterned side up, in the bottom of a plastic dish just larger than the master.  60- or 100-mm tissue culture dishes are well suited for this purpose.
3. In a polystyrene 50 ml centrifuge tube, combine the Sylgard components at a ratio of curing agent : elastomer base of 1 : 10 by weight.  Mix thoroughly using a plastic device, such as a disposable pipette. Prepare at least 0.2 ml of elastomer per square centimeter of dish area.
4. Centrifuge the 50 ml tube at 300 G for 5 min to remove air bubbles.
5. Pour the elastomer over the master, then place in a desiccator, under vacuum, for 30 minutes.
6. Cure the elastomer in an oven at 65°C for at least 2 hours. Curing at higher temperatures and for longer times results in stiffer elastomer.  Let cool to room temperature.
7. Separate the sheet of stamps from the master.

### 3. Microcontact printing of fibronectin onto glass

1. Cut out a single stamp. Stamps measuring 4 mm X 4 mm to 1 cm X 1 cm in area and 1 – 2 mm thick are easiest to start with.  Place pattern side up on a glass slide or small plastic dish.
2. Place stamp in a plasma cleaner, and process, under vacuum, for 30 seconds.  A Harrick Scientific plasma cleaner (see Equipment), set at its highest output setting will render the PDMS surface hydrophilic.  Longer times result in cracking of the elastomer.
3. Dilute the fibronectin solution with deionized water to a stamping concentration of 50 µg/ml.
4. Place a small drop (10 – 50 µl) of stamping solution on the stamp.  It will spread across the hydrophilic surface.  Add only enough solution that the drop covers the stamp, but does not run over the edges.  Let protein adsorb to stamp for 5 minutes.
5. Using a Kimwipe® or other clean paper tissue, wick off most of the protein solution from the stamp, without touching the patterned region.
6. Dry the remaining solution from the stamp under a stream of clean, dry, inert gas, such as nitrogen.
7. Using tweezers, remove the stamp from the glass slide, invert, and place in contact with the cleaned glass coverslip (the surface to be patterned).  Place a weight on top to promote good contact.  The specific weight that provides the best patterning is dependent on the stamp size and pattern; start with a 5  g weight, and adjust between stampings.  Leave stamp in contact with surface for 1 minutes.
8. Carefully disassemble the stack, and separate stamp from coverslip.
9. Vigorously rinse the patterned coverslip in PBS, followed by deionized water, to remove protein that is not adsorbed to the surface.  Dry coverslip under a nitrogen stream.

###  Representative Results

Microcontact printing is a powerful process for patterning molecules on surfaces.  This process has the ability to create features with dimensions ranging from tens of micrometers to hundreds of nanometers; in Fig. 1A, the logos on the left are each 200 µm in height, while the green spots illustrated in Fig. 1B are 1 µm in diameter and spaced at 4 µm intervals, measured center-to-center.  Fig. 1B also illustrates a powerful property of microcontact printing, namely that it is and additive process.  Multiple rounds of microcontact printing can be applied to a single surface to create multicomponent systems^3^.


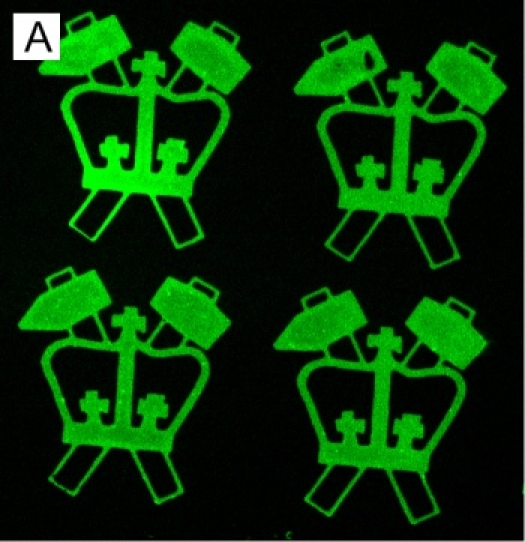
 
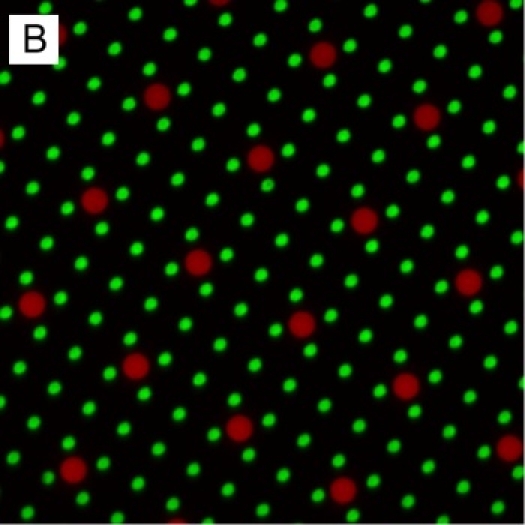


## Discussion

The microcontact printing process is conceptually simple and very robust, having been applied to patterning a wide range of molecules on a variety of substrates. However, this process remains something of an art. The specific geometry of the pattern to be created, protein to be patterned, applied weight, and coating/stamping conditions all affect the stamping quality. For example, too little weight, applied to large features, often results in gaps in the pattern as can be seen in the upper right logo of Fig. 1A. Conversely, too much weight will cause sagging and collapse of the stamp, resulting in unintended deposition of protein in regions between patterned features.As a second example, specific proteins (such as antibodies) pattern with better fidelity if the plasma treatment step is omitted, leaving the PDMS hydrophilic. The stamping of fibronectin onto glass is presented here as a starting point for such modifications and optimizations, demonstrating the basic techniques of this process.
